# TRUST IN THE ORGANIZATION AS A MEDIATOR IN THE RELATIONSHIP OF GOOD ORGANIZATIONAL CONTEXT WITH EMPLOYEE WELL-BEING

**DOI:** 10.13075/ijomeh.1896.02615

**Published:** 2025

**Authors:** Agnieszka Czerw, Damian Grabowski, Agata Chudzicka-Czupała, Katarzyna Stąpor

**Affiliations:** 1 SWPS University, Institute of Psychology, Faculty of Psychology and Law in Poznań, Warsaw, Poland; 2 SWPS University, Institute of Psychology, Faculty of Psychology in Katowice, Warsaw, Poland; 3 Silesian University of Technology, Gliwice, Poland

**Keywords:** stress at work, authentic leadership, well-being in the workplace, trust in organization, areas of worklife, propensity to trust

## Abstract

**Objectives:**

This study examined whether trust in supervisor, co-workers, and the organization mediates the relationship between organizational context and employee well-being. The research aimed to identify which components of trust have the strongest mediating effect, which organizational context elements are most strongly related to trust, and which dimensions of well-being are best explained by this model.

**Material and Methods:**

The study involved 1113 employees from various Polish organizations, averaging 45 years of age, with 41% having higher education. Participants completed questionnaires measuring areas of worklife, authentic leadership, trust propensity, trust in supervisors, trust in co-workers, trust in organization, workplace well-being, job satisfaction, and work stress. Structural equation modeling was used to analyze relationships between 3 latent variables: well-functioning organization (WFO), full trust in the organization (FTO), and well-being in the workplace (WB).

**Results:**

The best-fitting model showed that full trust in the organization completely mediates the relationship between a WFO and WB. The WFO explained 90% of the variance in FTO. The WFO most strongly explained trust in the organization as a whole (81%) and trust in supervisors (68%), with weaker explanation of trust in coworkers (37%). The FTO explained 87% of the variance in WB, which in turn was strongly associated with job satisfaction (70% of variance) and negatively with work stress (34% of variance).

**Conclusions:**

A well-functioning organization characterized by value congruence, fair rewards, recognition, authentic leadership, and supportive peer groups strongly influences full organizational trust, which in turn enhances well-being and job satisfaction while reducing work stress. Trust serves as a complete mediator between organizational context and employee well-being, with trust in the organization and in supervisors playing particularly important roles in this relationship.

## Highlights

- Trust fully mediates between organizational context and employee well-being.
- Institutional trust matters more than peer trust for workplace well-being.
- Value congruence drives trust more than rewards or fairness.

## INTRODUCTION

The purpose of the study presented here was to examine whether and to what extent trust in supervisor, co-workers and the organization is related to well-being in the workplace and good organizational context including job characteristics and leadership. More specifically, it was decided to see if the individual components of complete trust in the organization can be considered as a mediator in the relationship between good organizational context and employee well-being.

### Trust in the organization as a complex construct

Trust, as a psychological phenomenon, is a specific feeling towards an entity that is felt particularly strongly in a risky situation. It is the belief that the motives, attitudes and behaviors of another person, group of people or other defined entity are generally favorable and benevolent towards us [1,2]. Giving trust to others is important both in the context of private life and in the context of work, because in both contexts uncertain, risky situations are very common. Working people who want to achieve both their own and organizational goals must very often rely on others in the organization. Trust in an organization is a very complex phenomenon, because it can apply not only to individuals, co-workers, leaders, but also to entire teams, management or even the organization as an institution. It also refers to the trust of whole groups in other social groups – including organizational groups [3–5].

In general, there are 2 types of trust in a work situation: interpersonal trust and impersonal trust [2,6,7]. At the same time, interpersonal trust can also be analyzed from 2 perspectives: lateral trust in co-workers, and vertical trust in a superior [8, 9]. Impersonal trust is trust in the organization as a formal institution [2,6,10,11]. The 2 perspectives are related but are separate constructs [3,12]. Even trust in a supervisor and trust in an organization are separate psychological constructs, as evidenced by the results of studies showing the differential consequences of the 2 types of trust [3].

A review of the available literature indicates that authors tend to focus on one of these perspectives in their studies. More comprehensive studies simultaneously considering all types of trust in the work context are virtually non-existent. The authors were able to find 1 study carried out in 3 perspectives of trust: towards colleagues, towards superiors and towards the organization. However, in this study the measurement of trust was simplified to a single question asked directly about its level [13]. The study presented here uses measurement of 3 types or areas of trust: in the organization as a whole, in superiors and in co-workers. The authors believe that the research problem discussed in this article will help to broaden knowledge about the importance of trust in professional work.

### Trust in organizations – a review of research

The importance of organizational and intra-organizational trust is confirmed by numerous research findings. In the research projects encountered in the literature, trust in the organization happens to be both the dependent and independent variable. Most often, the importance of trust for the well-being of employees both in the context of work and from a life perspective is indicated.

Research confirms the importance of trust for employee well-being. A Canadian and U.S. study [14] found that trust in management and colleagues was more important for life and job satisfaction than financial aspects of work. Among police officers, interpersonal trust (in co-workers, immediate and senior superiors) positively correlated with job involvement, organizational commitment, and job satisfaction [9], with trust in colleagues being the strongest predictor of these attitudes. Similar positive associations between organizational trust (both lateral and horizontal and organizational attachment were confirmed in a study of >500 employees [8]. Similarly, a meta-analysis of a number of studies in the medical community confirms the importance of trust in leaders and in the organization for work engagement, cooperation in teams and other positive organizational behaviors [15].

Other studies have tested hypotheses about the determinants of trust in organizations. First and foremost, factors related to organizational culture have been pointed out as significant reasons for increasing trust in an organization [16]. This encompasses HR practices [17] designated as “high-involvement” practices, which enhance information flow and cultivate empowerment and employee engagement. These principles also encompass organizational principles such as relationship building, clear communication, effective training, competency management, and informal meeting opportunities [18]. On the other hand, practices related to cognitive bridging, emotional embodying and inclusive enacting are highlighted by researchers of organizations with the experience of facing a major financial crisis [19]. The authors of these studies point to the exceptional effectiveness of such activities in maintaining trust in organizations. In turn, changes inworking styles in recent years, such as the widespread introduction of remote work options in some industries, have also directed researchers' attention to the possible relationship between remote work and the level of trust in organizations. A study conducted during the COVID-19 pandemic made it possible to test this connection. It found that remote work was associated with an increase in trust in organizations, but not necessarily in co-workers [13].

Another strand of research on the determinants of trust in organizations focuses on the characteristics and behaviors of leaders or leadership styles. Already the classic article by Mayer et al. [1] pointed to such leader traits as ability, benevolence, and integrity, and later research confirms this and points to ability, integrity, fairness, and openness as the 4 key determinants of trust in leaders [20]. Sharing knowledge and information with employees is another important behavior [2]. Other studies conducted with qualitative methods on a group of health care workers [21] indicate the importance of such principles implemented in the organization as integrity/equity and consistency, reward and recognition, perceived respect from supervisor/manager, empowerment and autonomy, supervisor/manager encouragement of employee development. Perceived characteristics and behaviors of leaders are related to preferred leadership styles, among other things. In a study conducted during the COVID-19 pandemic among hospital employees [22], transformational leadership style was shown to play a significant role in building trust in the organization.

In addition to situational or organizational determinants of trust, it seems sensible, to take into account employee characteristics. Some studies point to the importance of the trusted person's organizational affiliation, hierarchical relationship (supervisor or peer), and gender compatibility particularly evident in the context of the initial level of trust for a new team member [23]. A separate issue seems to be the specific individual characteristic of personal level of trust. Propensity to trust, as a personal variable, is defined as a general disposition to trust others regardless of the situation [24]. Some studies on organizational trust include this variable in general research model. Early work on organizational trust already theorized this trait as one of the predictors of trust in organizations [1]. Similarly, Searle et al. [17] assumed that the level of employee propensity to trust is positively related to trust in the employer. They confirmed this assumption on a group of respondents from 41 countries. The relationships were admittedly not strong, but consistent. This pattern of relationships may explain the dependence of trust propensity levels on a country's culture. A series of surveys conducted on a multitudinous group of respondents from 36 countries showed significant associations of propensity to trust levels with collectivism. Significant differences were found not only between collectivist and individualist countries, but also in the relationship between personal appreciation of collectivistic values and levels of trust propensity. In each case, collectivism was positively associated with the trust propensity [24]. The results of another study [25] support the conclusions about the relationship between propensity to trust and employee behaviors such as risk-taking, citizenship or counterproductive behavior (downgrading), when controlling for the level of trust in the organization.

In Poland, there have been a trust problem for years. There is one of the lowest rates of social trust in Europe and the world. Cyclical surveys conducted in this area show that for years virtually nothing has changed [26] and only 1 in 5 respondents (19%) believes that most people can be trusted. On the other hand, the vast majority believes that one must be very careful in relationships with others (77%). For this reason, it is interesting to include this concept in the proposed research model. Trust propensity was also included in a study of trust in organizations in a Polish sample of employees. Lewicka [8] showed that it is positively related to horizontal and vertical interpersonal trust, and indirectly to attachment to the organization. This result is so promising that it also provides justification for including this variable in the presented research problem.

As a summary of the search for data on the determinants of trust in organizations, one can cite article Khouya and Benabdelhadi's [27] discussing a review of research on the subject. They point to 3 groups of determinants of trust in an organization: factors at the level of the individual (personality traits, propensity to trust), factors at the level of the organization (organizational support, organizational effectiveness and human resource policies) and factors at the level of culture (values, power distance, individualism/collectivism).

Since a number of studies have proven that trust can be both a dependent variable on other contextual factors (culture, organizational activities, job characteristics, etc.) and an explanatory variable for employee attitudes and behavior, so naturally the question of the possible mediating role of this construct arises. Such analyses have already been done. For example, a study by Kannan-Narasimhan and Lawrence [28] showed that trust in a supervisor mediated the relationship between behavioral integrity (the alignment an actor's words and deeds perceived by another person) and employee attitudes and behaviors such as organizational citizenship behavior, organizational commitment and organizational cynicism (negatively). This was particularly evident in the case of trust in senior management. Similar results were obtained in a study of nurses, in which trust in leaders mediates between transformational leadership style and nurses' innovative actions [29]. Another study worth mentioning is the one conducted on insurance agents in Taiwan [19]. This study found that trust in the organization mediated the relationship between trust in colleagues and organizational commitment and performance. This is an interesting result showing that different types of trust within an organization can have individual significance.

Another important starting point for our research problem was a meta-analysis of studies on trust in immediate supervisors as a mediator between job characteristics and attitudes, behaviors of employees [30]. As a result of a meta-analysis of many studies, the authors formulated the assumption that trust in immediate supervisors mediates between leaders actions and practices (e.g., leadership style, acquiescence participation in organizational decisions, or perceived organizational support), employee attitudes (e.g., trust propensity) and employee performance and behavioral outcomes, job attitudes and intentions. Another important study for the authors' assumptions is a project that analyzed in a group of university teachers the relationship between areas of worklife and job exhaustion and the mediating role of trust in organizations [10]. The study confirmed the mediation of trust in the organization for 4 of the 6 job characteristics (workload, fairness, reward, and value). This allows us to make a similar assumption about the mediating role of organizational trust between organizational context (measured by, among others, areas of worklife) and employee well-being.

Another study of the mediating role of trust between contextual factors and employee behaviors is worth mentioning. The study was conducted on a group of Chinese senior- or middle-level managers studying the Executive Master of Business Administration [31]. Contextual factors were understood as 3 levels of organizational functioning: leadership role (transformational leadership), structural rule (formalization, centralization) and cultural norm (business value, ethical value), and employee behaviors were in-role and extra-role performance. The authors confirmed their predictions almost completely. Trust in the organization mediated between transformational leadership style, business value, ethical value and employee behaviors. Also, a study conducted in a steel manufacturing organization [32] considered leadership style. It tested whether trust in the organization mediated between authentic leadership style and employee flourishing. This expectation was confirmed, which is another important starting point for us to build our own research model.

In the absence of a comprehensive theory linking trust types to organizational characteristics and employee well-being, our research synthesizes 3 key models of trust in organization: Mayer et al.'s [1] interpersonal trust dynamics, McKnight et al.'s [33] propensity to trust framework, and Vanhal's [34] interpersonal-impersonal trust distinction. This multi-model approach provides a foundation for examining trust as a mediator between organizational characteristics and employee well-being. The innovation of the authors' project lies in combining multiple levels of trust with different indicators of organizational characteristics and employee well-being.

### Research aims, questions and hypothesis

The aim of this research project was to understand how trust in an organization is related to its characteristics and the well-being felt by employees. To achieve this goal, several research questions and one hypothesis were formulated:

– question (Q)1: Is trust in the organization a mediator between the organizational context (characteristics of the organization) and employee well-being?
– hypothesis (H)1: Full trust in the organization (FTO) is a mediator in the positive relationship of organizational context called well-functioning organization (WFO) with employee's well-being (WB).

**Figure 1:**
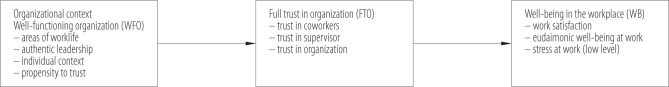
Research model: full trust in organization (FTO) as a mediator of organizational context (well-functioning organization – WFO) and individual context with employee well-being (WB), Poland, March 2021

It should be noted that all the variables included in the hypothesis are composed of several indicators. For this reason, they are also defined here. Full trust in the organization includes trust in the organization as an institution, trust in supervisors, and trust in coworkers; WFO is a sense that the organization is functioning smoothly and has a positive climate, represented by organizational features and an authentic leadership style; WB is represented by a high level of satisfaction and a low level of stress. The starting point was the following model shown in Figure 1. All components can also be found in Table 1.

**Table 1:** Components and levels of organizational context, full trust in organization and employee well-being, in the study among 1113 employees from various Polish organizations, Poland, March 2021

Variable	Component
Organizational context (well-functioning organization [WFO]) and individual context	– areas of worklife workload (low level) control reward community fairness values – authentic leadership (AL) self-awareness relational transparency internalized moral perspective balanced processing – propensity to trust (PT) (individual context)
Full trust in organization (FTO)	– trust in coworkers cognition-based trust in coworkers affect-based trust in coworkers – trust in supervisor skills and competencies kindness and integrity – trust in organization organizational transparency and kindness organizational certainty and ethics
Well-being in the workplace (WBS)	– positive organization – fit and development – positive relations with coworkers – contribution to the organization – work satisfaction – stress – low level or lack of stress

In addition, 3 other research questions were posed. Due to the lack of data to support any predictions, these were treated as exploratory questions and no hypotheses were made in relation to them:

– Q2: Which of the 3 levels of trust (in the organization as a whole, superiors, co-workers) marks the strongest in mediating between organizational context and well-being?
– Q3: Which components of the organizational context mark the strongest within the trust relationship?
– Q4: Which dimensions of organizational well-being, ill-being are most explained in this model?

It is worth noting that the above model also includes a variable from the individual context, namely propensity to trust.

## MATERIAL AND METHODS

### Sample description

In order to verify the research hypothesis, 1113 people working in various organizations across Poland were surveyed. The inclusion criteria for the sample comprised individuals who were >18 years old (of legal age in Poland) and who had accumulated a min. 12 months of professional experience. Respondents ranged in age from 18 to 77 years, with the middle 50% of respondents in the 33–57 years age range. The respondent's average age was almost 45 years (M±SD 44.98±14.08 years). The largest number of respondents had higher education (N = 452, 41%) and secondary education (N = 431, 39%), while the rest had bachelor's and engineering degrees (N = 147, 13%), vocational (N = 71, 6%) and elementary (N = 12, 1%).

The survey covered 241 employees of micro enterprises ≤9 employees (22% of the sample), 304 of small enterprises ≤49 employees (27%), 238 of medium-sized enterprises ≤249 employees (21%) and 330 of large enterprises ≥250 employees (30%). Representatives of various professions were surveyed, related to social sector work (helping professions and working with people, such as therapists, doctors, nurses, salespeople, customer advisors) and technical professions, related to information processing and agriculture (e.g., engineers, mechanics, construction technicians, IT specialists, economists). Most of the respondents, 957 people (86%), are contracted and employed full-time. The remaining 156 people (14%) were employed under civil law contracts, commission agreements and work contracts.

A group of 734 people were employed by private companies (66%), 319 by state institutions (29%), 31 by cooperatives (2.7%) and 29 by NGOs (2.3%). The average length of service was just over 22 years (M±SD 22.14±14.74 years). As many as 273 respondents held managerial positions (24.5%). Participation in the study was voluntary and anonymous. The research design was approved by the Research Ethics Committee of the Institute of Psychology at University SWPS (approval No. 2020-29-11). Data were collected using an online survey.

### Methods

The areas of worklife and perception of authentic leadership were assumed to be indicators of a broadly defined (good) organizational context including organizational climate and culture [35–37]. High scores on these scales signify a good and pleasant climate and a strong organizational culture, in short, a WFO, mainly in the social and psychological sense.

The *Areas of Worklife Scale* [35,37,38] has been adapted to Polish by Terelak and Izwantowska [37]. The scale consists of 29 items across 6 subscales (workload, control, reward, community, fairness, and values) using a 5-point Likert scale (α = 0.70–0.89, ω = 0.71–0.90).

The *Authentic Leadership Questionnaire* [36] is a tool designed to assess the qualities and behaviors associated with authentic leadership. The instrument comprises 4 subscales: self-awareness, relational transparency, internalized moral perspective, and balanced processing. It uses a 5-point Likert scale (α = 0.81–0.94, ω = 0.82–0.94).

The *Trust Propensity Scale* [39] is a measurement tool that assesses an individual's tendency to trust others. It is a 4-item scale with a single factor and a 5-point Likert answer scale (α = 0.89, ω = 0.88).

The *Trust in Supervisor Scale* [40] assesses the level of trust in a supervisor. It consists of 20 items and 2 subscales: skills and competence; kindness and integrity. A 5-point Likert scale was used (α = 0.92, ω = 0.92 for both subscales). The author of the questionnaire also considered using an overall summary score (α = 0.89, ω = 0.92). This indicator was used in this study.

The *Scale of Affect- and Cognition-Based Trust* [41,42] is a measurement tool that assesses the extent to which trust is influenced by emotional state and cognitive function. This scale comprises 11 items and 2 subscales (emotion-based trust and cognitive-based trust) and uses a 5-point Likert scale (α = 0.86–0.95, ω = 0.88–0.95).

The *Trust in the Workplace Questionnaire* (TWQ) by Czerw, Grabowski, and Chudzicka-Czupała was prepared for this study (for this reason, the description of this method has been expanded). The organization is understood as an institution, not the people who make it up. The questionnaire consists of 40 items, grouped into 2 scales.

Organizational transparency and kindness, is related to employees' beliefs that they are important to their organization, that the organization recognizes and cares about their needs and enters into open, honest communication with them (30 items; α = 0.98, ω = 0.98).

Organizational certainty and ethics, means the belief that the organization treats employees equally and fairly and sees and appreciates their commitment and effort at work. It is also a belief in adequate and legitimate organizational procedures that are used appropriately (10 items; α = 0.95, ω = 0.95).

In this research, it was decided to use the global score – the sum of all 40 items (α = 0.98, ω = 0.98), which is taken as an indicator of trust in organizations.

In the confirmatory factor analysis, the second order factor loaded the factors: organizational transparency and kindness, organizational certainty and ethics, with strengths of 0.76 and 0.89, respectively. And the model including only 1 first order factor obtained the following measures of fit: χ^2^(df) = 9481.77 (740), χ^2^/df = 8.73, root mean square error of approximation (RMSEA) = 0.083, standardized root mean square residual (SRMR) = 0.084, confirmatory fit index (CFI) = 0.984, normed fit index (NFI) = 0.982. It can be seen that measures such as SRMR, CFI, NFI indicate a satisfactory and good fit of the data to the model [43]. The most important indicator for the so-called one-dimensionality test, the SRMR value, fell within the range of satisfactory fit (p < 0.1) [44,45]. The above analytical results testify to the high reliability and accuracy of this questionnaire in its univariate version.

The *Workplace Well-Being Questionnaire* [46] is a tool designed to assess employees' psychological well-being in the workplace. It contains 43 items across 4 subscales: positive organization, fit and development, positive interpersonal relationships, and contribution to the organization. The items are rated on a 7-point Likert scale (α = 0.94–0.96, ω = 0.94–0.96).

The *Job Satisfaction Scale* [47] is a tool used to assess job satisfaction. The scale consists of 5 items, each measured using a 7-point Likert scale (α = 0.91, ω = 0.91).

The *Perceived Stress Scale* [48] assesses subjective stress experiences. The scale consists of 10 items, each measured using a 5-point Likert scale (α = 0.82, ω = 0.83).

### Statistical methods

To verify the hypothesis and research questions, correlation coefficients were calculated and structural equation modeling (SEM) was performed. Within the SEM several models were built taking into account 3 latent variables: WFO, FTO, and WB. The components of these variables are presented in Table 1. Statistical analyses were carried out using package the SPSS v. 27 statistical and JASP 0.18.3.

## RESULTS

The first step of the analysis was the calculation of correlation coefficients. Table 2 presents descriptive statistics, including means, standard deviations, distribution shape measures (skewness and kurtosis), as well as the results of the Shapiro-Wilk test used to assess the normality of the data distribution. As can be seen from Table 2, the distributions of all variables deviate from the normal distribution. The Shapiro-Wilk test is statistically significant for all the variables shown. Besides, the skewness divided by standard error of skewness and kurtosis divided by standard error of kurtosis are higher than the key value of 1.96 for most (17 and 10, respectively) of the variables [49]. Therefore, non-parametric statistics (Spearman correlations) and SEM with the unweighted least squares (ULS) estimator were employed for the verification of the hypothesis and research questions [50]. Table 3 contains correlations and intercorrelations between studied variables. As shown in Table 3, trust in supervisor, trust in co-workers and trust in organization correlate positively moderately and significantly with components of organizational well-being, satisfaction and negatively moderately (trust in coworkers) with stress experienced at work. Areas of worklife correlates positively, weakly (workload), moderately and significantly with levels of organizational trust (coworkers, supervisors and organization) and components of well-being and job satisfaction. Propensity to trust correlates weakly and positively almost with all variables in this study, only exception is workload (this correlation is very weak, but significant). This variable correlates most strongly with trust in coworkers, as well as elements of well-being (most strongly with positive relations with coworkers and contribution to the organization). Authentic Leadership correlates positively and on average level with all areas of worklife, all trust forms, and well-being. Thus, it can be concluded that the components of a WFO and positive climate (areas of worklife, authentic leadership) correlate positively with trust (and its levels coworkers, supervisors and organization) and well-being, satisfaction and negatively with stress. Also, trust and its levels correlate positively with well-being and satisfaction and negatively with stress. Thus, it can be predicted that trust, including all of its forms, is a mediator between WFO and well-being in the organization.

**Table 2:** Descriptive statistics characterizing the empirical distribution, along with the Shapiro-Wilk (SW) test for normality in the study among 1113 employees from various Polish organizations, Poland, March 2021

Variable	Min. [pts]	Max [pts]	M [pts]	SD [pts]	Skewness	Sk/SE of Sk	Kurtosis	K/SE of K	SW	p
1. Workload	6	30	19.38	4.52	–0.12	–1.68	0.01	0.10	0.99	<0.001
2. Control	3	15	11.07	2.16	–0.34	–4.68	0.33	2.25	0.96	<0.001
3. Reward	4	20	13.35	3.07	–0.06	–0.88	0.12	0.81	0.98	<0.001
4. Community	5	25	17.73	3.85	–0.49	–6.67	0.36	2.43	0.97	<0.001
5. Fairness	6	30	18.59	4.07	–0.36	–4.96	0.71	4.81	0.98	<0.001
6. Values	5	25	17.35	3.41	–0.21	–2.81	0.28	1.88	0.98	<0.001
7. Authentic leadership	16	80	53.12	11.16	–0.15	–2.11	0.49	3.33	0.99	<0.001
8. Trust in supervisor	22	100	69.95	17.30	–0.38	–5.16	–0.07	–0.48	0.98	<0.001
9. Trust in coworkers	12	55	39.21	8.02	–0.45	–6.18	0.56	3.82	0.97	<0.001
10. Trust in the organization	42	200	131.69	32.08	–0.20	–2.79	–0.05	–0.36	0.99	<0.001
11. Propensity to trust	4	20	14.32	3.13	–0.66	–9.05	0.73	4.99	0.95	<0.001
12. Positive organization	12	60	41.60	9.43	–0.39	–5.36	0.10	0.70	0.98	<0.001
13. Fit and development	10	50	36.60	7.70	–0.47	–6.48	0.41	2.76	0.97	<0.001
14. Positive relations with coworkers	13	65	48.18	9.11	–0.48	–6.53	0.69	4.72	0.97	<0.001
15. Contribution to the organization	8	40	29.91	5.66	–0.55	–7.53	0.94	6.41	0.96	<0.001
16. Work satisfaction	5	35	23.37	6.01	–0.53	–7.27	0.37	2.52	0.97	<0.001
17. Work stress	10	45	26.59	6.08	–0.51	–7.01	–0.11	–0.71	0.96	<0.001

SE of K – standard error of kurtosis; SE of Sk – standard error of skewness; K/SE of K – kurtosis divided by standard error of kurtosis; Sk/SE of Sk – skewness divided by standard error of skewness.

SE of Sk = 0.07, SE of K = 0.15.

**Table 3:** Correlations and intercorrelations between components of well-functioning organization (WFO) (perception of worklife areas, authentic leadership), levels of trust (trust in coworkers, trust in supervisors and trust in the organization), propensity to trust, components of well-being in the workplace (work satisfaction and work stress) in the study among 1113 employees from various Polish organizations, Poland, March 2021

Variable	Correlation																
	1	2	3	4	5	6	7	8	9	10	11	12	13	14	15	16	17
1. Workload	–																
2. Control	0.30	–															
3. Reward	0.34	0.48	–														
4. Community	0.25	0.46	0.60	–													
5. Fairness	0.26	0.43	0.61	0.54	–												
6. Values	0.25	0.54	0.61	0.59	0.66	–											
7. Authentic leadership	0.17	0.39	0.51	0.50	0.61	0.59	–										
8. Trust in supervisor	0.25	0.44	0.59	0.57	0.65	0.66	0.72	–									
9. Trust in coworkers	0.12	0.33	0.39	0.58	0.36	0.47	0.40	0.49	–								
10. Trust in the organization	0.27	0.45	0.64	0.58	0.77	0.75	0.68	0.78	0.45	–							
11. Propensity to trust	0.09	0.31	0.23	0.36	0.21	0.33	0.25	0.29	0.38	0.27	–						
12. Positive organization	0.24	0.47	0.63	0.59	0.69	0.73	0.67	0.75	0.49	0.89	0.32	–					
13. Fit and development	0.15	0.47	0.58	0.55	0.51	0.64	0.53	0.60	0.56	0.68	0.37	0.81	–				
14. Positive relations with coworkers	0.20	0.44	0.56	0.73	0.53	0.63	0.57	0.65	0.65	0.71	0.42	0.81	0.79	–			
15. Contribution to the organization	0.13	0.45	0.49	0.54	0.43	0.56	0.44	0.53	0.55	0.58	0.38	0.72	0.80	0.77	–		
16. Work satisfaction	0.22	0.46	0.59	0.51	0.54	0.63	0.57	0.61	0.48	0.69	0.34	0.79	0.80	0.69	0.64	–	
17. Work stress	–0.48	–0.38	–0.50	–0.45	–0.40	–0.48	–0.28	–0.46	–0.35	–0.49	–0.20	–0.48	–0.42	–0.45	–0.40	–0.45	–

All correlation coefficients are significant at the p < 0.01 level.

**Figure 2:**
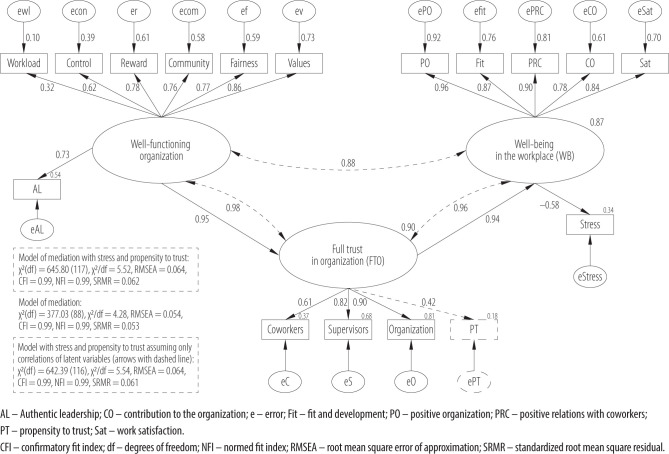
Results of structural equation modeling: full trust in organization (FTO) as mediator in the relationship between organizational context (FTO) and employee well-being and job satisfaction (well-being in the workplace – WB) in the study among 1113 employees from various Polish organizations, Poland, March 2021

To show this mediation structural equation modeling was used. Models were built with 3 latent variables: WFO, FTO, and WB. The ULS estimator was used in the analysis. This is because it was assumed that the questionnaire response scales were ordinal scales, and it was noted that the distribution of data within scales was asymmetric (Table 2) [49,50]. The models that contain significant paths between each of the explicit and latent variables are shown in Figure 2.

The best-fitting models turned out to be those in which complete trust in the organization is a complete mediator in the positive relationship between a WFO and wellbeing in the organization. Well-being, on the other hand, correlates strongly and positively with work satisfaction and negatively and quite significantly with work stress.

The relationship of a WFO to well-being occurs through trust in the organization involving all 3 levels. It can be assumed that a WFO determines trust in the organization, which in turn is an important factor shaping well-being. The variable WFO explains the following explicit variables: most strongly values (the similarity of the values important for the organization and for the employee) (73% of the variance of this variable), reward (the belief that the company rewards its employees well and cares about employees, and satisfies the need for recognition and respect) (61% of the variance), fairness (the belief, that the organization treats the employee fairly) (59% of the variance), community (sense of support, togetherness, belief that colleagues can be counted on) (58% of the variance), the authentic leadership (54% of the variance) and control (the extent to which the employee decides scope of the tasks performed and on the manner of their performance) (39% of the variance). This variable explains to a lesser extent the feeling of workload, or rather the lack of it (only 10% of the variance) but as much as 90% of the variable FTO.

The variable FTO explains the following explicit variables: trust in the organization most strongly (81% of the variance) and trust in supervisors (68% of the variance), more weakly trust in coworkers (37% of the variance). The weakest FTO explains propensity to trust (only 18%). Full trust in the organization explains as much as 87% of the variance of WB.

The variable WB explains the following explicit variables: the strongest positive organization (92% of variance), positive relations with coworkers (81% of variance), fit and development (76% of variance), work satisfaction (70% of variance), slightly weaker contribution to the organization (61% of variance) and the weakest low work stress (34% of variance).

The model, which takes into account all of the abovementioned variables (model of mediation with stress and propensity to trust) obtained a satisfactory and good fit to the data with the exception of the ratio χ^2^/df, which clearly exceeded the value of 3, i.e., the limit of the satisfactory fit interval (2< χ^2^/df <3), χ^2^(df) = 645.80 (117), χ2/df = 5.52, RMSEA = 0.064, CFI = 0.988, NFI = 0.985, SRMR = 0.062 [38]. Propensity to trust and workload are the least explained in this model. Removing these variables from the model improves model fit (Δχ^2^ = 268.77, p < 0.001) and the values of the fit indices are as follows: χ^2^(df) = 377.03 (88), χ^2^/df = 4.28, RMSEA = 0.054, CFI = 0.993, NFI = 0.990, SRMR = 0.053. In addition, propensity to trust was introduced as a component of full trust in the organization and not as a component of WFO (this model was a worse fit).

A very similar fit to the model including all variables, was obtained by the model with all variables assuming only correlations between the 3 latent variables (arrows sketched with a dotted line in Figure 2), i.e., WFO, FTO and WB (χ^2^(df) = 642.39 (116), χ^2^/df = 5.54, RMSEA= 0.064, CFI = 0.988, NFI = 0.985, SRMR = 0.061).

## DISCUSSION

In summary, a WFO relating primarily to the compatibility of employee and organizational values, the satisfaction of the need for recognition and respect, fair rewards linked to authentic leadership and support within the employee group is strongly associated with full trust in the organization comprising mainly trust in the company as a whole and trust in superiors and, to a lesser extent, trust in colleagues. Full trust, on the other hand, appears as a possible factor that increases well-being and satisfaction with the organization and work, and as a variable that reduces work stress. Thus, based on the above analyses, trust can therefore most likely be viewed as a total mediator in the relationship of organizational efficiency (good climate) with well-being. This model was the only one to include all relevant pathways. It should be noted, however, that this is not the only model for explaining organizational reality. In fact, the areas of good functioning, trust and well-being are strongly intertwined and even form an inseparable whole, as has been shown again by the model that assumes only correlations between the 3 variables. Less significant factors in the relationship with trust are a sense of lack of workload, a sense of control, a sense of lack of strain and stress, and trust in colleagues. The propensity to trust is also less pronounced in this relationship, which is consistent with the results of previous studies [39].

A good organizational context (WFO) is therefore an important element in human work, linked to both well-being and trust. It can be surmised with high probability that a positive context determines a high level of trust, which then enhances employee well-being. A positive context of work environment and well-being means a smoothly functioning organization in which there is congruence between employee and organizational values, efficient and fair rewards, satisfaction of the need for recognition and respect linked to authentic leadership and the occurrence of supporting employee groups. A positive context also means a good organizational climate and a strong culture [51].

The analyses presented in this article have clearly shown that 3 areas are strongly interrelated: the context of the organization, trust in and within the organization, and wellbeing. A good climate most likely implies higher levels of trust (primarily in the organization and in superiors), while the latter may determines well-being (i.e., the perception of the organization as a positive place, positive evaluation of relationships with co-workers and a sense of fit) and satisfaction, which is associated with low level of stress experienced by employees.

As the above analyses show, an important element of the organizational context is the compatibility of the organization's values with those of its employees. Values define what a person considers important in his or her daily life and help shape behavior. This also takes place in the organization's environment. In organizational theory, values are usually conceptualized as fundamental elements of organizational culture and as instruments for setting goals, influencing people, and as an important tool for leadership [52,53]. This understanding of values remains fundamental to management theory. Successful organizations communicate the meaning of values, priorities and ways of doing things in a clear, concise and commercial way, so that every employee understands them and can contribute to the achievement of common goals, which become a kind of signpost for them. Also, other studies [54] indicate that values prevalent in the workplace can inspire, encourage and enable employees to contribute effort and creativity, promote individual development. This, in turn, affects, on the one hand, the productivity and profitability of the entire organization and, on the other hand, the healthy functioning at work of the employees of such an organization

It is worth remembering that this also has an impact on the employee's identity. It helps them to build their self-image as a person. This is proven by our research. Since values influence both emotions and behavior, they can serve as a kind of individual compass for the employee. The important role of an employee's sharing of meaningful values (core values) by the organization where he or she is employed is also confirmed by the research of other authors. Guillemin et al. [55] emphasize that values, not only ethical, but also cultural and social, give meaning to people's lives and work, allow them to realize their passions, feel commitment while doing their jobs. The support of an employee's values by the organization raises his sense of dignity and is associated with an increase in the experience of respect. The realization of important values has a positive impact on the well-being and health of employees by creating a healthy workplace. Awareness of the importance of one's values and the degree to which they are respected in the organization, is becoming more widespread, which translates into opportunities to honor and protect each employee and help them experience their core values through work.

Similarly, satisfying the need for recognition and respect from co-workers, support and interest from managers, care and rewards received proved to be important. This is also confirmed by studies by other authors. For example, it has been shown that respect in the workplace and support from managers is related to organizational identification [56]. According to the aforementioned research the effect of workplace respect and managerial support on organizational identification is mediated by the level of interpersonal trust between co-workers and job satisfaction.

Other researchers also have demonstrated that support from managers can not only affect employee well-being, but it also promotes trust among co-workers in general [57]. Earlier studies that focused on social exchange relationships between employees and supervisors [58] indicate that if employees are satisfied with their managers, the decisions they make, and at the same time managers provide them with support when needed, this can allow to develop trust among employees. When employees perceive the organizational climate as fair, respectful and trustworthy, this usually also results in a real increase in trust and greater job satisfaction, and ultimately profits the entire organization.

It is also worth noting that in addition to the ontological perspective, the epistemological perspective related to the measurement of the phenomena described above is important. Namely, the measurement of organizational context and well-being often include items similar to those used in the study of trust and trust is sometimes even an indicator of climate [35,51]. This certainly strengthens the relationships between the variables shown here. However, it does not mean that these variables are not truly related [37]. Especially since variables such as rewards (reward) devoid of trust items also correlate quite strongly with trust.

### Limitations and future studies

While the study provides valuable insights into the relationship between organizational characteristics, trust, and employee well-being, it has limitations that should be acknowledged. First, since it was self-descriptive and questionnaire-based, it would be worthwhile for future research to consider other approaches, including qualitative methods, to deepen our understanding of the relationships between the studied variables. The main shortcoming of this study is its cross-sectional nature; all variables were examined at a single point in time. Therefore, future research should examine variables at 3 different points in time, with a significant gap between each point. First, examine context; then, trust; and finally, well-being. Based on the presented studies, the authors can conclude that the context of the organization, trust within and in the organization, and well-being are strongly interrelated. However, the mediation model should be regarded as more of a hypothesis.

Secondly, the survey was conducted among people working in different organizations across the country with different characteristics and structures. The employees performed different tasks and occupied different positions within their organizations. This makes it difficult to determine the importance of the organizational conditions and the role of the organizational structure in the results obtained. In future studies, it would be worthwhile to better control and take into account relevant organizational features and characteristics when making comparisons among employees working in different types of organizations. However, this research has the advantage of a large sample size that resembles the working population of Poland because quota sampling was used.

### Practical implications

The presented findings suggest that aligning employee values with those of the organization is a key element of effective organizational functioning. This requires clear communication of the organization's goals, priorities, and operational principles so every employee can identify with and act in accordance with them. Organizations should foster a culture of authentic leadership, fair rewards, and team support, as this promotes full trust in the organization and its leaders. Such trust translates into greater well-being, job satisfaction, and lower stress levels among employees. From a management perspective, these findings highlight the importance of investing in hard structures and systems, as well as in shaping the organizational climate, building a culture of collaboration, and developing leadership competencies [59,60]. Implementing these practices can reduce turnover, increase motivation, and improve mental health. Organizations should regularly monitor levels of trust and wellbeing through surveys to allow for swift responses to potential threats to organizational effectiveness. Finally, creating a work environment in which values are reflected in daily operations contributes to developing a sustainable and healthy workplace.

## CONCLUSIONS

The objective of this study was to investigate the correlation between organizational characteristics, trust, and employee well-being. The findings indicated that a WFO is significantly associated with the presence of complete trust within the organization. This, in turn, functions as a total mediator that enhances eudaimonic well-being and job satisfaction while reducing work-related stress. The study identified key elements of a WFO that contribute to trust. These elements include the alignment of employee and organizational values, the recognition and respect of employees' needs, fair reward systems, and support within employee groups, all of which are linked to authentic leadership. The study further established that trust in the organization manifests primarily as trust in the organization as an institution and trust in superiors, while trust in colleagues plays a less prominent role. The article emphasizes the significance of shared values as fundamental components of organizational culture. The article emphasizes that organizations which clearly communicate their values enable employees to make a meaningful contribution to shared goals. This, in turn, supports the formation of employees' identities and serves as an individual compass for their behavior. The primary advantage of this research project is its comprehensiveness. Each latent variable is composed of multiple observable variables, which makes it possible to understand the importance of trust in an organization at multiple levels. Consequently, the practical implications of improving trust and employee wellbeing can also be addressed to very specific organizational processes as well as to a more general understanding of organizational culture.
